# Efficacy and safety of ruxolitinib for graft-versus-host disease prophylaxis in allogeneic hematopoietic stem cell transplantation: a systematic review and meta-analysis

**DOI:** 10.3389/fmed.2025.1680188

**Published:** 2025-11-26

**Authors:** Chunhong Xie, Lingling Shi, Zhenbin Wei, Yongrong Lai, Rongrong Liu

**Affiliations:** Department of Hematology, The First Affiliated Hospital of Guangxi Medical University, Nanning, Guangxi, China

**Keywords:** ruxolitinib, GVHD, prophylaxis, allo-HSCT, meta-analysis

## Abstract

**Introduction:**

Graft-versus-host disease (GVHD) remains a major complication after allogeneic hematopoietic stem cell transplantation (allo-HSCT). Despite standard prophylaxis, acute and chronic GVHD incidence remains high. Ruxolitinib, a Janus kinase inhibitor (JAK1/2), shows promise in treating steroid-refractory GVHD. However, its efficacy and safety as an adjunct to standard prophylactic regimens remain subjects of debate. This meta-analysis aims to evaluate the efficacy and safety of ruxolitinib when used as an adjunct to GVHD prophylaxis following allo-HSCT.

**Methods:**

Comprehensive studies were searched in PubMed, Web of Science, Embase, Cochrane Library, and ClinicalTrials.gov. Outcomes measures included the incidence rates of acute/chronic GVHD, overall survival (OS), cytomegalovirus (CMV) and Epstein-Barr virus (EBV) reactivation events. Pooled proportions with 95% confidence intervals (CIs) were calculated using random/fixed-effects models.

**Results:**

A total of 12 studies, including 406 patients, were analyzed. Most of these studies were non-randomized. The pooled incidence of grade II–IV and III–IV acute GVHD was 10.4% (95% CI: 7.3–13.5%) and 2.9% (0.6–5.2%), respectively, with no heterogeneity (*I*^2^ = 0%). Chronic GVHD occurred in 26.8% (19.2–34.4%). One- and two-year OS rates were 86.6% (78.8–94.5%) and 81.2% (68.2–94.2%). CMV and EBV reactivation rates were 30.6% (14.6–46.6%) and 19.0% (0.4–37.7%), respectively.

**Discussion:**

Ruxolitinib as GVHD prophylaxis significantly reduces acute GVHD severity and maintains favorable survival outcomes, likely due to Janus kinase and signal transducer and activator (JAK-STAT) pathway inhibition. However, elevated CMV/EBV reactivation rates the need for vigilant monitoring. These findings support ruxolitinib’s role as a promising adjunct in GVHD prevention, warranting further randomized trials to confirm long-term safety and efficacy.

## Introduction

Allogeneic hematopoietic stem cell transplantation (allo-HSCT) is a potentially curative treatment for hematological malignancies, bone marrow failure syndromes, and inherited disorders (1). The therapeutic efficacy of allo-HSCT is partly attributed to the graft-versus-leukemia (GVL) effect, wherein donor immune cells recognize and eliminate residual malignant cells in the recipient, thereby reducing the risk of disease relapse (2). However, this benefit is often counterbalanced by the development of graft-versus-host disease (GVHD), which remains a leading cause of non-relapse mortality and significantly impacts the patient outcomes (3, 4).

Preventing GVHD requires a delicate balance between suppressing alloreactive immune responses to mitigate tissue damage and preserving immune reconstitution to maintain the GVL effect. Currently, the most widely used GVHD prophylaxis involves the use of calcineurin inhibitors (CNIs) (e.g., cyclosporine or tacrolimus) combined with methotrexate (MTX) or mycophenolate mofetil (MMF) with or without the addition of an antithymocyte globulin (ATG) product (5), and posttransplant cyclophosphamide (PTCy). Despite the extensive use of prophylaxis regimens, acute GVHD occurs in 30–50% of transplant recipients, with severe forms (grade III–IV) developing in about 15% of transplant recipients (4). Additionally, chronic GVHD develops in 30–40% of patients, with a substantial proportion experiencing moderate to severe forms that impair long-term quality of life and survival (3, 6).

Recent advances in understanding the pathophysiology of GVHD have highlighted the critical role of the Janus kinase (JAK)–signal transducer and activator (STAT) of the transcription pathway. This pathway regulates the differentiation of T cells (7) and the production of inflammatory cytokines (8), which contribute to tissue inflammation and damage. Ruxolitinib, an oral JAK1/2 inhibitor, has demonstrated efficacy in the treatment of both acute and chronic steroid-refractory GVHD (9, 10). Preclinical studies have demonstrated that ruxolitinib effectively treats steroid-resistant GVHD while maintaining the GVL effect, with a favorable safety profile characterized by mild side effects (11, 12). Building on this success, clinical trials have begun to explore its potential as a prophylactic agent. Kroger et al. first demonstrated the efficacy of ruxolitinib in preventing acute GVHD in patients with myelofibrosis undergoing allo-HSCT (13). Their study revealed a markedly low incidence of grade II-IV acute GVHD at 8%, contrasting with a significantly elevated cytomegalovirus (CMV) reactivation rate of 41% in this cohort. Contrasting these findings, Hong et al. documented a 22.2% cumulative incidence of acute GVHD in ruxolitinib-treated patients, coupled with only 11.1% demonstrating clinically relevant CMV reactivation (14). Recently, Wu et al. established the efficacy of a low-dose ruxolitinib prophylactic regimen [combined with cyclosporine (CSA) and MTX] in achieving a marked reduction of grade II–IV acute GVHD, with an incidence rate of 7.8% (15).

Existing studies evaluating ruxolitinib as an adjunct to GVHD prophylaxis after allo-HSCT have produced conflicting results, primarily due to limitations such as single-center designs and small sample sizes. These inconsistencies prevent definitive conclusions about its role in standard prophylaxis regimens. To address this, we conducted a systematic review and meta-analysis to assess the efficacy and safety of ruxolitinib as an adjunct to GVHD prophylaxis following allo-HSCT.

## Methods

This meta-analysis was based on the Preferred Reporting Items for Systematic Reviews and Meta-analysis (PRISMA) (16).

### Search strategy and selection criteria

This investigation implemented a systematic literature retrieval across four major electronic databases (PubMed, Web of Science, Embase, and Cochrane Library) and ClinicalTrials.gov, encompassing all English-language studies indexed through 31 January 2025. The following string was used to perform the literature search: (ruxolitinib OR JAK inhibitor) AND (graft-versus-host disease OR graft-versus-host disease OR GvHD OR aGvHD OR cGvHD). The detailed search strategy of PubMed is shown in Supplementary Table S1. The references of included articles were also searched to assay additional studies. Inclusion criteria were: (1) clinical trials investigating evaluating the efficacy of ruxolitinib for the prevention of GVHD in populations that underwent allo-HSCT, (2) Cases with > 5 patients, (3) studies with consistent criteria of observation items, and (4) studies reported a quantitative outcome of interest. We excluded individual case reports, reviews, comments, editorials, and studies that did not report a quantitative outcome of interest. Two separate researchers (C. Xie and L. Shi) conducted the literature search and determined study eligibility. In cases of disagreement, they deliberated on their points of view, and if consensus could not be reached, a third reviewer (R. Liu) was brought in to assist.

### Data extraction

Two reviewers (C. Xie and L. Shi) independently assessed the titles and abstracts based on the inclusion criteria. Any discrepancies were resolved by involving a third reviewer (R. Liu). Afterward, they conducted a full-text review of the literature to make the final determination of eligibility. We used a standardized extraction form to extract information about the name of the first author, study design, year of publication, number of patients, age, gender, disease characteristics, treatment regimens, the dosage of ruxolitinib, and outcome parameters during the follow-up period. The outcome parameters comprised the incidence and severity of acute GVHD, chronic GVHD, overall survival (OS), CMV, and Epstein-Barr virus (EBV) reactivation.

### Quality assessments and certainty of the evidence

The risk of bias in the included studies was assessed using Methodological Index for Non-Randomized Studies (MINORS) (17). This index consists of 12 items that examine critical aspects such as study design, sample size, outcome assessment, statistical analysis, and control for confounding factors. Each study was scored on a scale of 0–2 for each item, with a total score out of 16 (applicable for non-comparative studies). The evaluation standards were as follows: a score between 0 and 8 indicated low quality; a score between 9 and 12 signified moderate quality; and a score ranging from 13 to 16 reflected high quality.

The quality of the evidence was evaluated utilizing the Grading of Recommendations, Assessment, Development, and Evaluations (GRADE) approach (18), which considers five key factors: (1) risk of bias, (2) consistency, (3) indirectness, (4) imprecision, and (5) publication bias. The evidence was classified into categories of high, moderate, low, or very low quality. Modifications to the grade were implemented based on the methodological rigor of the studies, allowing for potential downgrading or upgrading of the evidence classification. Two authors (C. Xie and L. Shi) worked independently on methodological quality assessment and GRADE evaluation. If disagreements occurred, they deliberated on their points of view, and if consensus could not be reached, a third reviewer (R. Liu) was brought in to assist.

### Statistical analysis

Data processing and statistical analysis were conducted using R software (version 4.4.2). A Cochran *Q*-test and *I*^2^ statistic were used to investigate heterogeneity. The pooled event proportions with their respective 95% confidence intervals (CIs) were calculated using a random or fixed-effects model. When heterogeneity was significant (*p* < 0.10 or *I*^2^ > 50%), a random effects model was adopted to pool the results. The meta-analysis results were visually presented using forest plots; if possible, with heterogeneity further investigated through subgroup and sensitivity analyses. The potential publication bias was scrutinized utilizing Egger’s test, with a *p* > 0.05, suggesting an absence of significant publication bias.

## Results

### Study selection and characteristics

**FIGURE 1:**
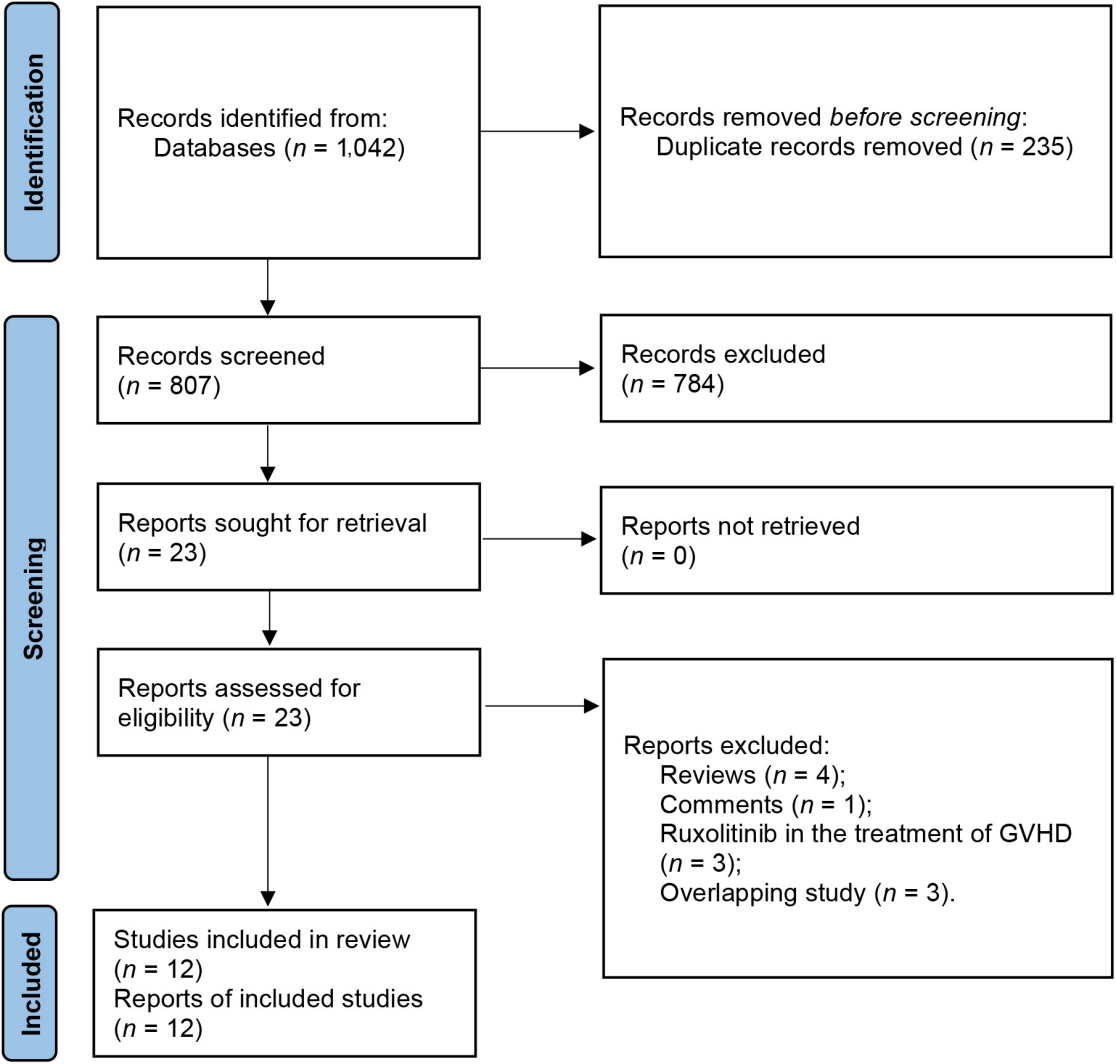
Preferred Reporting Items for Systematic Reviews and Meta-analysis (PRISMA) flow diagram of the included studies.

We initially retrieved 1,042 articles that appeared to be eligible from electronic databases. After eliminating 235 duplicates, 807 records were assessed based on their titles and abstracts, and those not meeting the inclusion criteria were excluded. The remaining 23 studies were then evaluated by reviewing their full texts. As a result, 11 studies were excluded, comprising 12 studies that fulfilled the inclusion requirements (13–15, 19–27). The selection process is illustrated in Figure 1.

The characteristics of the included studies were summarized in Table 1. Twelve studies with 406 patients were included in this meta-analysis. All studies were single-arm clinical trials except three. The dosage of ruxolitinib ranged from 5 to 20 mg daily. The median time to neutrophil engraftment ranged from 10 to 27 days and was predominantly 11–15 days. The median time to platelet engraftment ranged from 13 to 38 days and was predominantly 13–15 days.

**TABLE 1:** Characteristics of included studies.

References	Study design	Number of patients	Median age	Sex (M/F)	Diagnosis	Stem cell sources	HLA compatibility/ donor	Ruxolitinib (mg/day)	Conditioning regimen	GVHD prophylaxis	Neutrophil engraftment (days)	Platelet engraftment (days)
Abedin et al. (19)	Prospective phase II study	20	67 (61–78)	NR	AML/MDS	PB	Matched RD or UD	10	RIC	PTCy + TAC + MMF	13 (12–15)	13 (10–25)
Ali et al. (20)	Prospective phase I study	18	65 (25–73)	14/4	Myelofibrosis	PB	Matched sibling or UD	10 or 20	RIC of Flu/Mel	TAC + SIR	17 (12–23)	25 (13–119)
Chen et al. (21)	Retrospective study	22	2.5–13	10/12	Thalassemia	NR	Matched UD, Haplo	5	Bu/CY + Flu + ATG	CSA + MMF + MTX	10 (9–12)	13 (11–16)
Cheng et al. (22)	Retrospective study	8	20.5 (14–58)	6/2	ALL	NR	Matched sibling or UD, Haplo	10–20	TBI + CY + etoposide; cytarabine + Bu/Cy	CSA or TAC + ATG + MTX + MMF	NR	NR
Defilipp et al. (23)	Prospective phase II study	63	68 (61–79)	NR	AML/MDS	PB	Matched RD or UD	20	RIC of Bu/Mel	TAC + MTX	NR	NR
Hobbs et al. (24)	Prospective phase II study	43	66 (46–75)	27/16	Myelofibrosis	PB	Matched RD or UD	NR	RIC of Flu/Mel	TAC + MTX	15 (4–38)	25 (11–145)
Hong et al. (14)	Retrospective study	27	4 (3–7)	11/16	Thalassemia	NR	Matched UD, mismatched UD, Haplo	5	Bu/CY + Flu + ATG	CSA + MMF + MTX	11 (11–12)	15 (14–17)
Kroger et al. (13)	Retrospective Study	12	63 (43–71)	7/5	Myelofibrosis	PB	Matched sibling or UD, mismatched UD	10	Flu/Bu	CSA + MMF + ATG	12 (11–18)	NR
Morozova et al. (25)	Prospective study	20	51 (32–64)	10/10	Myelofibrosis	PB/BM	Matched sibling or UD, mismatched UD, Haplo	15	RIC of Flu/Bu	PTCy	27 (18–44)	38 (15–219)
Wu et al. (15)	RCT	103	12+	NR	Hematological malignancies	NR	Haplo	5 or 10	Bu/Cy + ATG	CSA/MTX	12 (9–21)	13 (6–28)
Zhang et al. (26)	Prospective study	41	28 (1–56)	25/16	ALL/AML	PB	Matched UD, mismatched UD, Haplo	Adults: 5 mg/day, children: 0.07–0.1 [mg/(kg/day)]	Bu/Cy; TBI/Cy	PTCy + ATG + CSA + MMF	13 (11–20)	15 (12–34)
Zhang et al. (27)	Retrospective study	35	27 (12–57)	16/19	Aplastic anemia	PB	Matched sibling, Haplo	10	Bu/CY + Flu + ATG	CSA or TAC + MTX	14 (10–24)	23 (8–112)

ALL: acute lymphoblastic leukemia; AML: acute myeloid leukemia; ATG, anti-thymocyte globulin; Bu: busulfan; CSA: cyclosporine; CY: cyclophosphamide; Flu: fludarabine; Haplo: haploidentical; MDS: myelodysplastic syndromes; Mel: melphalan; MMF: mycophenolate mofetil; NR: Not reported; PB: peripheral blood; PTCy: Post-transplant cyclophosphamide; RD: related; RIC: reduced intensity conditioning; Sir: sirolimus; TAC: tacrolimus; TBI: total body irradiation; UD: unrelated.

### Risk of bias in the selected studies

The quality assessment of the included studies was listed in Supplementary Table S2. All studies stated a clear aim, included consecutive patients, and had appropriate endpoints and follow-up period. The median MINORS score was 12 (range, 10–18), indicating fair-quality evidence (maximum score was 18).

### Incidence of GVHD

**FIGURE 2:**
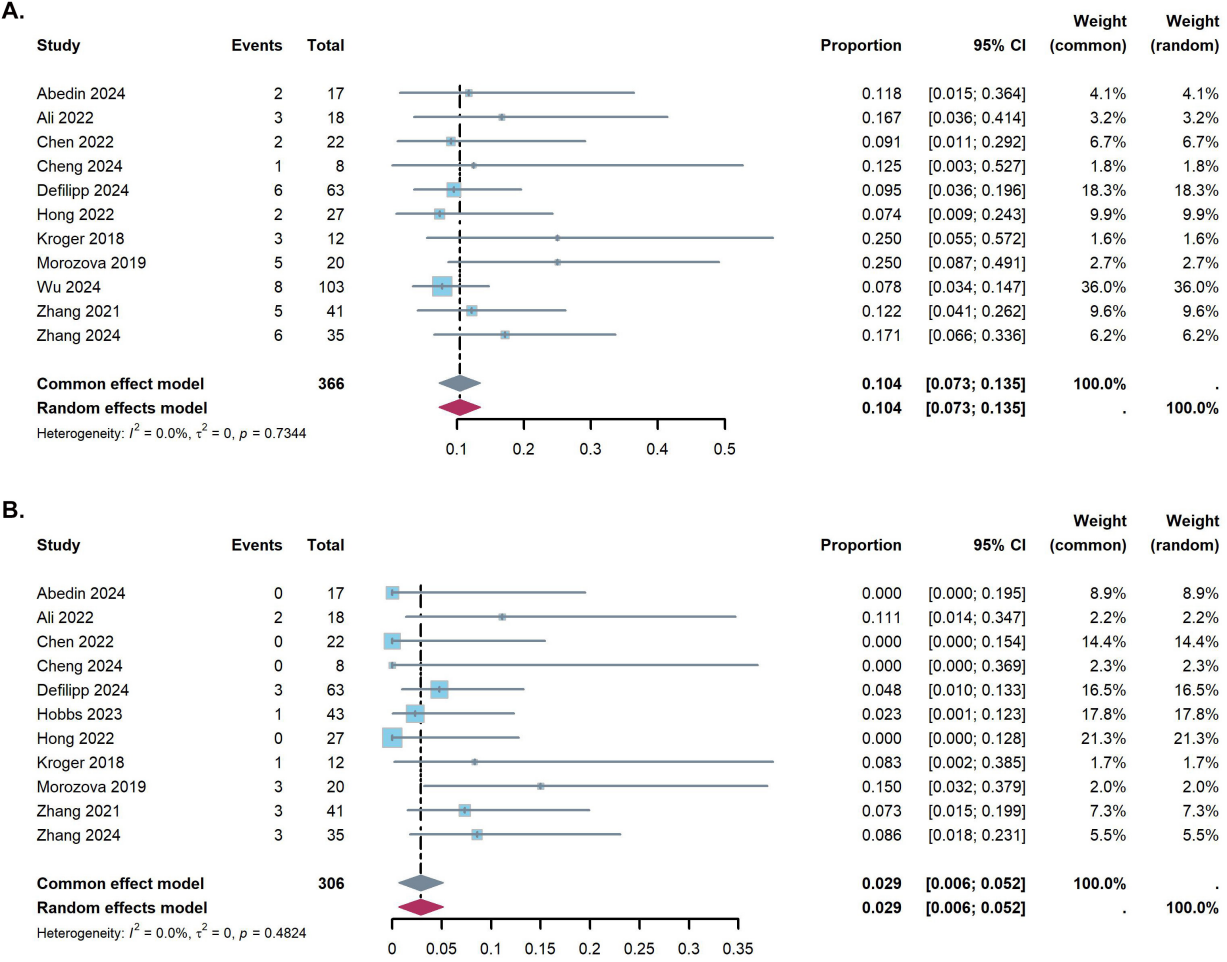
Forest plots for the pooled incidence of acute graft-versus-host disease [**(A)** grade II–IV acute graft-versus-host disease, **(B)** III–IV acute graft-versus-host disease].

The pooled results of grades II–IV acute GVHD and III–IV acute GVHD are shown in Figure 2. The probability rate of II–IV acute GVHD and III–IV acute GVHD after ruxolitinib for the prophylaxis was 10.4% (95% CI: 7.3–13.5%) and 2.9% (95% CI: 0.6–5.2%), respectively. There was no significant heterogeneity between the studies (acute GVHD grades II–IV: *I*^2^ = 0%, *p* = 0.734; acute GVHD grades III–IV: *I*^2^ = 0%, *p* = 0.482).

**FIGURE 3:**
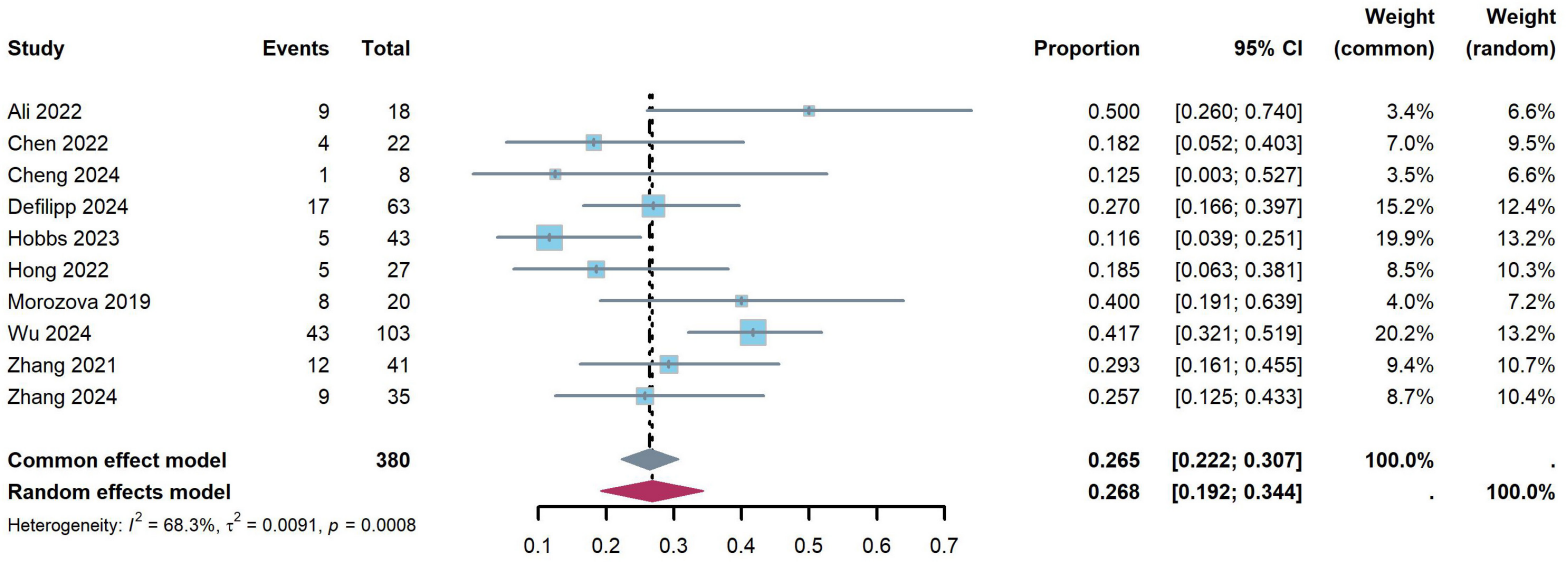
Forest plots for the pooled incidence of chronic graft-versus-host disease.

The incidences of chronic GVHD between 11.6 and 50.0% among the included 10 trials, and the pooled probability rate was 26.8% (95% CI: 19.2–34.4%), and heterogeneity between the studies was considered moderate (*I*^2^ = 68.3%, *p* = 0.001) (Figure 3).

### OS

**FIGURE 4:**
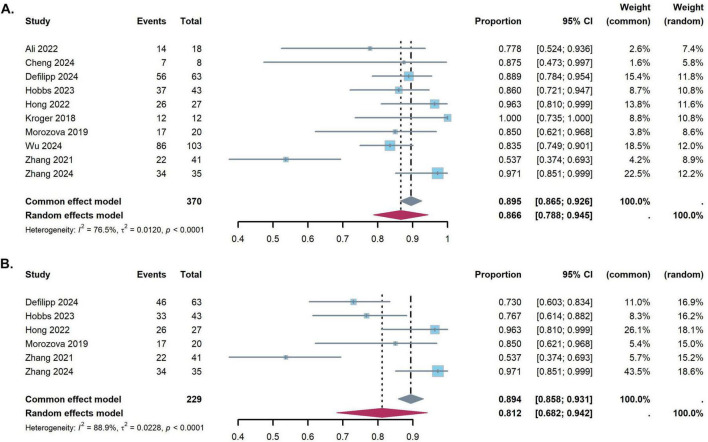
Forest plots for the pooled incidence of overall survival **(A)** 1-year overall survival, **(B)** 2-year overall survival).

One-year OS were reported in 10 studies, and 2-year OS were reported in six studies, respectively. The pooled results of 1-year OS and 2-year OS were 86.6% (95% CI: 78.8–94.5%) and 81.2% (95% CI: 68.2–94.2%), respectively. There is moderate heterogeneity between the studies in 1-year OS and 2-year OS (1-year OS: *I*^2^ = 76.5%; 2-year OS: *I*^2^ = 88.9%) (Figure 4).

### Incidence of CMV and EBV reactivation

**FIGURE 5:**
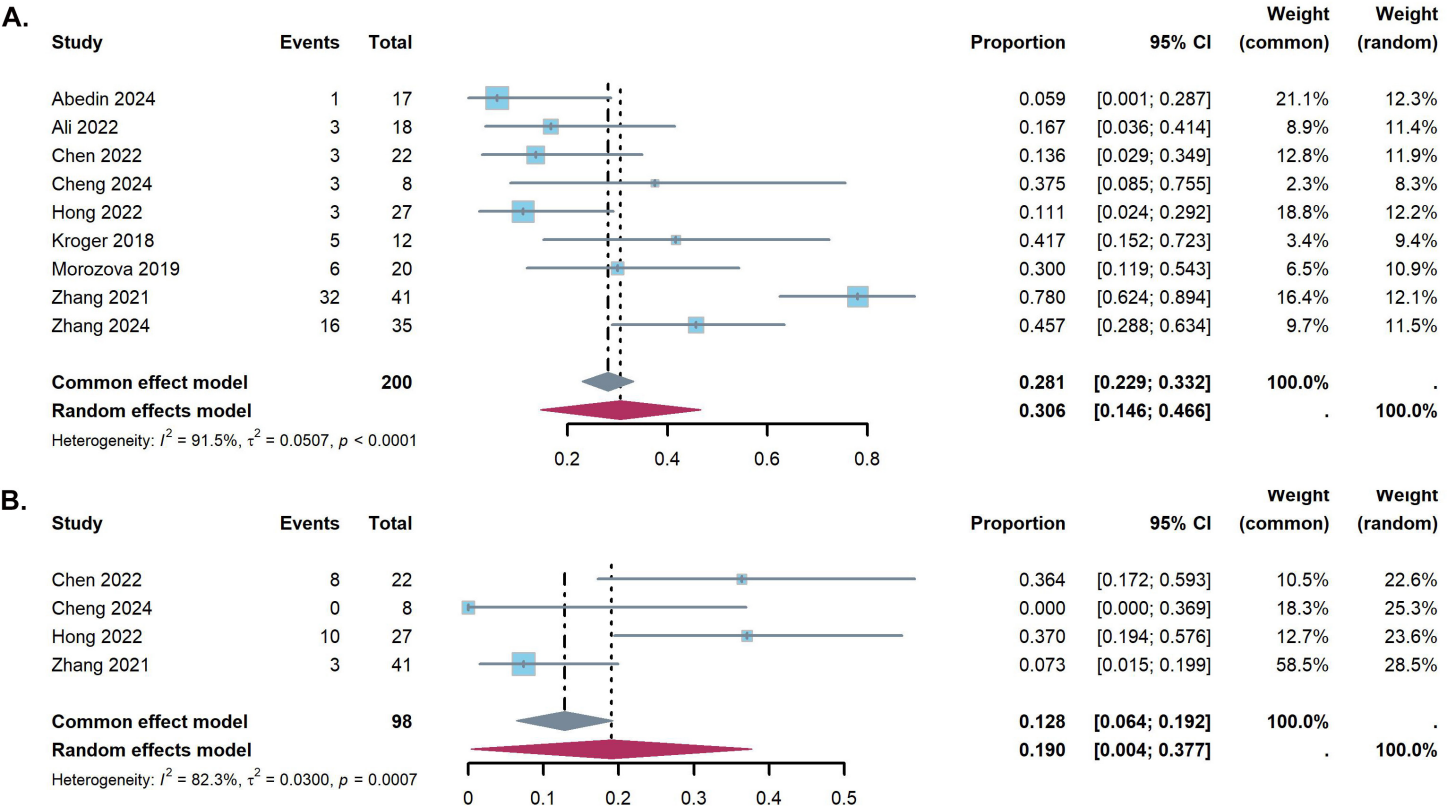
Forest plots for the pooled incidence of virus reactivation [**(A)** Cytomegalovirus reactivation, **(B)** Epstein-Barr virus reactivation].

The study revealed significant heterogeneity in CMV infection rate (*I*^2^ = 91.5%, *p* < 0.001). Random models were applied pooled results of CMV infection rate were 30.6% (95% CI: 14.6–46.6%) (Figure 5A). The pooled incidences of EBV infection were 19.0% (95% CI, 0.4 to 37.7%) (Figure 5B).

### Publication bias analysis

An analysis of publication bias was executed on the incorporated studies utilizing the Egger test. The results revealed that grades II–IV acute GVHD (*p* = 0.054), III–IV acute GVHD (*p* = 0.057), cGVHD (*p* = 0.800), and 1-year OS (*p* = 0.182) conformed to the criterion of *p* > 0.05. This implies that no significant publication bias exists within the study. Due to the small number of studies (less than 10), Egger’s test could not be performed for 2-year overall survival (OS), CMV, and EBV reactivation.

### Summary of quality of evidence

A summary of the results of a meta-analysis of within-group differences in the overall quality of evidence for the GRADE assessment is shown in Supplementary Table S3. Due to a potential bias in the single-arm test and heterogeneity, the evidence for chronic GVHD, 1-year OS, 2-year OS, incidence of CMV and EBV reactivation were rated as very low, while both grade II–IV acute GVHD and III–IV acute GVHD was scored as low-level evidence.

## Discussion

This meta-analysis of 12 studies involving 406 patients evaluated the efficacy and safety of ruxolitinib as an adjunct to GVHD prophylaxis following allo-HSCT. The pooled incidence of grades II–IV and III–IV acute GVHD was 10.4 and 2.9%, respectively, while chronic GVHD occurred in 26.8% of patients. One- and two-year OS rates were 86.6 and 81.2%, suggesting favorable outcomes. However, CMV and EBV reactivation rates were notably elevated at 30.6 and 19.0%. These findings suggest that while ruxolitinib is effective in preventing GVHD and improving survival, its potential to induce viral infections requires careful consideration.

Current GVHD prophylaxis mainly uses CNI regimens or PTCy protocols. Prata et al. found a 23% incidence of grades II–III acute GVHD at day 100, and 10% for limited chronic GVHD at 2 years in severe aplastic anemia patients with PTCy (28). Marco-Ayala et al. also showed consistent outcomes of 23% for grades II–IV acute GVHD and 28% for moderate-to-severe chronic GVHD (29). The study by Mehta et al. indicated higher grades II–IV acute GVHD rates with PTCy (52%) compared to Tac/MTX (42%), but reported similar rates of severe acute GVHD and chronic GVHD between the two treatments (30). A Swedish trial showed no significant differences in acute GVHD incidence between tacrolimus (TAC) and mycophenolate mofetil (Tac/MMF) and cyclosporine and mycophenolate mofetil (CSA/MMF) (5). Our meta-analysis indicates improved outcomes with ruxolitinib, showing a 10.4% incidence of grades II–IV acute GVHD and a 2.9% rate for grades III–IV, while chronic GVHD rates were 26.8%. The results highlight ruxolitinib’s significant role in reducing acute and chronic GVHD incidence when added to standard prophylaxis, likely due to its immunosuppressive effects via JAK-STAT pathway inhibition. The median times for neutrophil (10–27 days) and platelet (13–38 days) engraftment align with previous studies (31–34), indicating that ruxolitinib does not cause significant delays in myeloid or megakaryocytic reconstitution post-allo-HSCT. However, the integration of any novel agent, such as ruxolitinib, into standard care must consider practical implications beyond efficacy. The oral administration of ruxolitinib is a logistical advantage. But, its associated costs and the requirement for intensified viral monitoring represent significant economic considerations for healthcare systems. The favorable survival outcomes observed here suggest a potential clinical benefit that may offset these costs, but formal cost-effectiveness analyses are needed to confirm this.

Our meta-analysis demonstrates that the addition of ruxolitinib to GVHD prophylaxis yields pooled 1-year and 2-year OS rates of 86.6% and 81.2%, respectively, which compare favorably with the outcomes from CNI-based or PTCy–based monotherapy regimens. For instance, standard CNI-based regimens (e.g., CSA/MTX or CSA/MMF) achieve 2-year OS rates of 70–85% in matched donor transplants (34, 35), though with substantial variability depending on conditioning intensity and donor type. In contrast, phase 3 trials of PTCy–based regimens in non-haploidentical settings report 1-year OS rates of 75–88%, with superior GVHD control compared to CNI/MTX (76.8 vs. 72.6%) (36). Notably, the addition of ruxolitinib seems to enhance survival, potentially through its targeted immunosuppressive action, which may offer an advantage over the broader immunosuppressive effects of CNI and PTCy regimens. This suggests that ruxolitinib may provide a more effective strategy for improving survival in patients undergoing allo-HSCT.

The potent immunosuppressive effect of ruxolitinib, achieved through inhibition of the JAK-STAT pathway critical for T-cell and dendritic cell function (37), raises legitimate concerns about the increased risk of infections. Our meta-analysis revealed notable CMV and EBV reactivation rates of 30.6% and 19.0%, respectively. Although these reactivation rates are notable, it is crucial to interpret these findings in the appropriate clinical context. First, the reactivation rates are comparable to or even lower than those associated with PTCy-based regimens (42–69%) (38–40). Moreover, the included studies primarily reported laboratory-defined reactivation. Data on progression to clinically significant end-organ disease (e.g., CMV pneumonitis and EBV-posttransplant lymphoproliferative disorder), associated morbidity, and attributable mortality were limited, preventing robust pooling. Therefore, clinical and laboratory indicators signaling increased viral reactivation require careful monitoring. However, current evidence does not confirm that ruxolitinib prophylaxis results in more severe viral disease compared to standard prophylaxis. Future studies should focus on relevant infection outcomes.

When contextualizing ruxolitinib within the standard GVHD prophylaxis landscape, its role can be further defined by comparison with other novel agents (e.g., abatacept, vedolizumab, and sirolimus) (41–43). Ruxolitinib distinguishes itself as an oral JAK1/2 inhibitor. It potentially provides broader suppression of the cytokine-driven inflammation central to GVHD pathogenesis. However, direct comparative studies with these agents are lacking. The choice among them may depend on donor type, risk profile, and a careful balance among mechanism-specific efficacy, toxicity, and cost. Therefore, head-to-head trials are needed to optimize their use. Furthermore, the interpretation of our findings on chronic GVHD must consider the varied clinical management across transplant centers, particularly in tapering CyA based on individual factors like chimerism and minimal residual disease. This variability, often unreported, could confound results, as aggressive tapering may affect chronic GVHD incidence and severity. However, the consistently low acute GVHD rates highlight ruxolitinib’s strong prophylactic effect.

## Limitations

This meta-analysis presents several limitations. First, although 12 studies were included, the overall sample size is relatively modest, which may limit the statistical power for certain outcomes. Second, the GRADE ratings were low to very low due to the characteristics of the included single-arm trials and the presence of statistical heterogeneity, which may reduce the confidence in the estimated effects. Third, while pediatric patients were included in some studies, insufficient data prevented separate analyses of adult and pediatric populations, possibly introducing bias. Fourth, we observed notable heterogeneity (e.g., *I*^2^ > 60%) in several pooled estimates, particularly for chronic GVHD, OS, and viral reactivation rates. However, the limited number of studies, we were unable to perform meaningful subgroup analyses based on donor type (matched vs. haploidentical), conditioning intensity, concurrent prophylaxis (PTCy vs. CNI-based) or hematologic diagnoses (e.g., acute lymphoblastic leukemia, myelofibrosis, and aplastic anemia). Consequently, the pooled estimates for these outcomes should be interpreted with caution, as the heterogeneity may compromise the precision of our summary statistics. Finally, data on relapse outcomes were reported limitedly, which hindered the analysis of ruxolitinib’s impact on disease relapse. Despite these limitations, the consistency of primary outcomes–particularly the reduction in acute GVHD incidence with low heterogeneity–remains robust across studies and aligns well with existing literature.

## Conclusion

In conclusion, the addition of ruxolitinib to GVHD prophylaxis following allo-HSCT effectively reduces both acute and chronic GVHD incidence, with favorable 1-year and 2-year survival rates. Ruxolitinib demonstrates enhanced efficacy compared to standard prophylactic regimens, likely due to its immunosuppressive effects through JAK-STAT pathway inhibition. However, the potential risk of CMV and EBV reactivation requires careful monitoring. These findings support ruxolitinib as a promising adjunct for GVHD prevention. However, the limitations of the included studies warrant further prospective, randomized, multicenter trials to confirm its long-term safety and efficacy, with stratification by donor type and underlying disease.

## Data Availability

The original contributions presented in this study are included in this article/Supplementary material, further inquiries can be directed to the corresponding author.
