# Supplementary material for: Efficacy and safety of ruxolitinib for graft-versus-host disease prophylaxis in allogeneic hematopoietic stem cell transplantation: a systematic review and meta-analysis
Source: Front Med (Lausanne). 2025 Nov 26;12:1680188. doi: 10.3389/fmed.2025.1680188 (PMC12690610; doi:10.3389/fmed.2025.1680188)
Supplement: Supplementary file 1 [file Supplementary_file_1.docx]

Supplementary Material

# Supplementary Tables

**Table S1 Search strategy in** **PubMed database**

| Search number | Search Details |
| --- | --- |
| 1 | "graft vs host disease"[MeSH Terms] |
| 2 | "graft versus host disease"[Title/Abstract] OR "graft versus host disease"[Title/Abstract] OR "graft vs host disease"[Title/Abstract] OR"GvHD"[Title/Abstract] OR "aGvHD"[Title/Abstract] OR "cGvHD"[Title/Abstract] |
| 3 | 1 OR 2 |
| 4 | "janus kinase inhibitors"[MeSH Terms] |
| 5 | "ruxolitinib"[Title/Abstract] OR "jak inhibitor"[Title/Abstract] OR "janus kinase inhibitors"[Title/Abstract] |
| 6 | 4 OR 5 |
| 7 | 3 AND 6 |

**Table S2. Literature quality assessment based on the Methodological Index for Non-Randomized Studies (MINORS).**

| MINORS |  |  |  |  |  |  |  |  | Suitable for comparison | | | |  |
| --- | --- | --- | --- | --- | --- | --- | --- | --- | --- | --- | --- | --- | --- |
| study | A stated aim of the study | Inclusion of consecutive patients | Prospective collection of data | Endpoint appropriate to the study aim | Unbiased evaluation of endpoints | Follow-up period appropriate to the major endpoint | Loss to follow up not exceeding 5% | Prospective calculation of the sample size | A control group having the gold standard intervention | Contemporary groups | Baseline equivalence of groups | Statistical analyses adapted to the study design | total |
| Abedin 2024 | 2 | 2 | 2 | 2 | 0 | 2 | 2 | 0 |  |  |  |  | 12 |
| Ali 2022 | 2 | 2 | 2 | 2 | 0 | 2 | 2 | 0 |  |  |  |  | 12 |
| Chen 2022 | 2 | 2 | 2 | 2 | 0 | 2 | 2 | 0 |  |  |  |  | 12 |
| Cheng 2024 | 2 | 2 | 0 | 2 | 0 | 2 | 2 | 0 |  |  |  |  | 10 |
| Defilipp 2024 | 2 | 2 | 2 | 2 | 0 | 2 | 2 | 0 |  |  |  |  | 12 |
| Hobbs 2023 | 2 | 2 | 2 | 2 | 0 | 2 | 2 | 0 |  |  |  |  | 12 |
| Hong 2022 | 2 | 2 | 0 | 2 | 0 | 2 | 2 | 0 |  |  |  |  | 10 |
| Kroger 2018 | 2 | 2 | 0 | 2 | 0 | 2 | 2 | 0 |  |  |  |  | 10 |
| Morozova 2019 | 2 | 2 | 2 | 2 | 0 | 2 | 2 | 0 |  |  |  |  | 12 |
| Wu 2024 | RCT |  |  |  |  |  |  |  |  |  |  |  | - |
| Zhang 2021 | 2 | 2 | 0 | 2 | 0 | 2 | 2 | 0 | 2 | 2 | 2 | 2 | 18 |
| Zhang 2024 | 2 | 2 | 0 | 2 | 0 | 2 | 2 | 0 | 2 | 0 | 2 | 2 | 16 |

**Table S3. The quality of evidence was assessed using the GRADE approach.**

| **Quality assessment** | | | | | | | **Effect** | | **Quality** | **Importance** |  |
| --- | --- | --- | --- | --- | --- | --- | --- | --- | --- | --- | --- |
|  |  |  |  |  |  |  |  |  |  |  |  |
| **No of studies** | **Design** | **Risk of bias** | **Inconsistency** | **Indirectness** | **Imprecision** | **Other considerations** | **No. of events/ Total** | **Proportion (95% CI)** |  |  |  |
| **Grade II–IV acute GVHD** | | | | | | | | | | |  |
| 11 | observational studies | Not serious | Not serious | Not serious | Not serious | none | 43/366 | 10.4% (7.3%–13.5%) | ⊕⊕OO LOW | CRITICAL |  |
| **Grade III–IV acute GVHD** | | | | | | | | | | |  |
| 11 | observational studies | Not serious | Not serious | Not serious | Not serious | none | 16/306 | 2.9% (0.6 to 5.2%) | ⊕⊕OO LOW | CRITICAL |  |
| **Chronic GVHD** | | | | | | | | | | |  |
| 10 | observational studies | Not serious | serious^1^ | Not serious | Not serious | none | 113/380 | 26.8% (19.2 to 34.4%) | ⊕OOO VERY LOW | CRITICAL |  |
| **1-year OS** | | | | | | | | | | |  |
| 10 | observational studies | Not serious | serious^2^ | Not serious | Not serious | none | 311/370 | 86.6% (78.8 to 94.5%) | ⊕OOO VERY LOW | IMPORTANT |  |
| **2-year OS** | | | | | | | | | | |  |
| 6 | observational studies | Not serious | serious^3^ | Not serious | Not serious | none | 178/229 | 81.2% (68.2 to 94.2%) | ⊕OOO VERY LOW | IMPORTANT |  |
| **CMV infection rate** | | | | | | | | | | |  |
| 9 | observational studies | Not serious | very serious^4^ | Not serious | Not serious | none | 72/200 | 30.6% (14.6 to 46.6%) | ⊕OOO VERY LOW | IMPORTANT |  |
| **EBV infection** | | | | | | | | | | |  |
| 4 | observational studies | Not serious | serious^5^ | Not serious | Not serious | none | 21/98 | 19.0% (0.4 to 37.7%) | ⊕OOO VERY LOW | IMPORTANT |  |

^1^ Heterogeneity between the studies was considered moderate
^2^ Heterogeneity between the studies was considered moderate
^3^ Heterogeneity between the studies was considered moderate
^4^ Heterogeneity between the studies was considered significant heterogeneity
^5^ Heterogeneity between the studies was considered moderate
